# Microhardness, Surface Roughness, and Wear Resistance Enhancement of Reinforced Conventional Glass Ionomer Cement Using Fluorinated Graphene Oxide Nanosheets

**DOI:** 10.1055/s-0044-1785188

**Published:** 2024-05-17

**Authors:** Mona R. Aboelwafa, Sarah D. Shaheen

**Affiliations:** 1 Department of Conservative Dentistry, Faculty of Dentistry, Sinai University, Kantara, Ismailia, Egypt; 2 Department of Operative Dentistry, Faculty of Oral and Dental Surgery, Misr University for Science and Technology, Cairo, Egypt

**Keywords:** fluorinated graphene oxide, conventional glass ionomer cements, Vickers' hardness numbers

## Abstract

**Objectives** Conventional glass ionomer cements (GICs) have been considered the most prevalent restorative material however; the reduced mechanical qualities and decreased wear resistance have been the main challenges facing their wide clinical application. This study was designed to assess the mechanical properties of fluorinated graphene (FG) oxide-modified conventional GIC.

**Materials and Methods** Composites of FG/GIC samples were prepared using (Medifil from PROMEDICA, Germany, shade A3) at different concentrations (0wt%) control group and (1wt%, 2wt% and 3wt% FG) groups using cylindrical molds (3mm × 6mm). FG was prepared using hydrothermal technique and characterized using XPERT-PRO Powder Diffractometer system for X-ray diffraction analysis and JEOL JEM-2100 high resolution transmission electron microscope. Vickers' hardness and wear resistance of GI samples were measured. Mechanical abrasion was performed via three-body tooth brushing wear test using ROBOTA chewing simulator coupled with a thermocycling protocol (Model ACH-09075DC-T, AD-Tech Technology Co., Ltd., Leinfelden-Echterdingen, Germany).

**Statistical Analysis** Comparisons between groups with respect to normally distributed numeric variables were performed using one-way analysis of variance test followed by posthoc test. While paired *t* -test was utilized for comparing data within the same group.

**Results** : The surface roughness values of GICs (1wt% FG) and (2wt% FG) composites were significantly lower than those of the control and 3wt%FG groups. Vickers' hardness numbers were significantly higher in FG/GICs composites than in the control group ( *p* ≤0.05).

**Conclusion** GIC/FG combinations have sufficient strength to resist the occlusion stresses with improved hardness as compared with conventional GIC. GIC/FG appeared to be a promising restorative material.

## Introduction

Glass ionomer cement (GIC) is commonly utilized in restorative dentistry, particularly for base under restorations, luting cement, restoring both permanent, deciduous teeth, and using minimally invasive restorative approaches. Its widespread use can be related to its strong chemical bonding, self-adhering, convenient filling, affordability, reduced thermal expansion coefficient, and substantial fluoride-releasing qualities. But its low wear resistance, brittleness, and weak crack propagation resistance limit its applications. 1 2 3

Various attempts have been applied to enhance its properties, including addition of metallic particles, microfibers, and resins that resulted in reduction in fluoride release and the cement biocompatibility. Moreover, zinc and chlorhexidine are utilized to enhance antibacterial GICꞌs qualities. 4 5 6

Recently, nanotechnology has been applied to dental materials with the goal of enhancing several their properties. Incorporation of 2 wt% reduced graphene silver nanoparticles into conventional GIC greatly improved the antibacterial properties, whereas the surface hardness and mechanical qualities were severely deteriorated; while adding zinc oxide nanoparticles decreased the GIC microhardness without significant enhancement in antibacterial activity. Halloysite nanotubes had boosted the GIC hardness and wear resistance, whereas fluoride release was decreased. 7 8 9 Conventional GIC modified using 1 to 4wt% forsterite nanoparticles reported high compressive strength, however, exhibited decreased bioactivity compared with conventional GIC. 10 One of the promising nanoparticles is graphene. Further, chemical alteration is necessary to synthesize graphene derivatives. 11 12 13

Graphene and its derivatives possess numerous implementations in various branches of dentistry, science, and technology owing to their unique chemical and physical qualities. They have been used in association with membrane for bone grafting, titanium dental implants, as well as for improving the dental products of teeth whitening. Concerning graphene's applications in dental restoratives, it improved the bioactivity, strength, hardness, and mechanical attributes of nanocomposites and dental adhesives. 14 15 16 17

Fluorinated graphene (FG), an emerging addition to the graphene derivative family, is a type of material that is only one molecule thick and has numerous distinctive properties. It is commonly utilized in material synthesis and offers advantages including antibacterial properties, good stability, low surface energy, excellent wear resistance, and outstanding mechanical strength. Recent studies indicate that incorporation of FG could significantly reduce the wear volume and maximize microhardness in composite materials. Also, the coefficient of friction is reduced with increasing FG content, resulting in improved biocompatibility. 18 19 20 21 22

Our research was designed to evaluate the impact of several concentrations of FG on surface roughness, wear resistance, and microhardness of conventional GIC. The tested hypothesis was that the mechanical attributes of conventional GIC could be boosted through the incorporation of FG nanosheets.

## Materials and Methods

A commercially available conventional glass ionomer restorative material (Medifil from PROMEDICA, Germany, shade A3) was used. The glass ionomer powder was blended with fluorinated graphene oxide (FGO) nanosheets prepared at Nano Gate Company (Cairo, Egypt) with different percentages (1wt%, 2wt%, and 3wt %).

## Preparation of Fluorinated Graphene Oxide

The hydrothermal method was used for FG preparation. Under magnetic stirring, ultrapure water (80 mL) was used to disperse GO powder (100 mg), and the dispersion underwent ultrasonic treatment for 60 minutes. Thereafter, it was then centrifuged to eliminate unsolvable ingredients. The GO solution was then magnetically stirred, while 40 wt% of hydrofluoric acid (10 mL) and 65 wt% of nitric acid (10 mL) were added. The resulting slurry was put into an autoclave lined with Teflon and kept for 12 hours at 180°C. Following the reaction, the obtained pure solution was immediately boiled in an oil bath to evaporate water, achieving white fluorinated graphene powder. 23

## Characterizations

### Size and Shape

The crystalline structure of synthesized FG was examined using JEOL JEM-2100 high-resolution transmission electron microscope (TEM) utilizing an acceleration voltage of 200 KV.G.

### XRD Analysis

A XPERT-PRO Powder Diffractometer system was used to create an X-ray diffraction (XRD) pattern, with parameters of 2 Θ (20–80 degrees), a minimum step size of 2 Θ: 0.001, and wavelength (Kα) = 1.54614 degrees.

## Fabrication of Fluorinated Graphene/Glass Ionomer FG/GICs Disks

Various GIC/FG composites were produced using mechanical mixing technique. FG with varied concentrations of 1wt%, 2wt%, and 3wt%, respectively, were first ultrasonically disseminated in anhydrous ethanol (10mL), and subsequently, a specified quantity of GIC powder was incorporated into the dispersion. After ultrasonication for 1 hour, the consistent mixture was put in an agate mortar and properly ground till all of the ethanol had evaporated. Considering the manufacturer's directions, the resulting powder was blended with GICꞌs liquid in a 3:1 solid-to-liquid mass ratio. 23

Samples were formed into cylindrical molds with dimensions of 3mm thickness and 6mm diameter. The molds were loaded with the mixture and coated with a glass slide that was gently smoothed by manual pressing so the air voids in the cement paste were removed. Samples were kept for 24 hours at a humidity level of 100% at 37°C. Before testing, specimens were smoothed and polished using sandpapers of different sizes. A total of 40 samples, 10 for each group ( *n* = 10), were prepared for microhardness measurement and wear resistance assessment. The GIC/FG samples with various FG concentrations were identified as followed; GIC (0 wt% FG), GIC (1 wt% FG),GIC (2 wt% FG), and GIC (3 wt% FG). The GIC (0 wt% FG) represented the control group, while the remaining groups served as the experimental ones. Specimens with surface defects or pores were discarded. 23 24

## Wear Resistance Test

Mechanical abrasion was applied by three-body tooth brushing wear testing through utilizing the newly designed, programmable, four-station multimodal ROBOTA chewing simulator* coupled with a thermocyclic protocol powered by a servo-motor (Model ACH-09075DC-T, Ad-Tech Technology Co., Ltd., Germany).

The samples were placed in Teflon housings in the lower sample holder with cyanoacrylate glue. A loading of 300 g of brushing force, which is comparable to 3 Newton, was exerted. The test was applied as an equivalent to a 1-year clinical simulation (30400 strokes) of brushing conditions, with a slurry produced by blending calcium carbonate dentifrice and distilled water in a 2:1 ratio utilizing an electrical toothbrush (D12.013, Oral B, Germany). 25

## Surface Roughness Analysis

Optical techniques are often ideal for non-contact quantitative surface topography characterization. At a specific magnification of 120X, each sample was photographed using a USB digital microscope with an integrated camera (U500X Capture Digital Microscope, Guangdong, China) linked to a suitable computer.

The area of roughness measurement was standardized and specified by cropping the digital microscope images to 350 × 400 pixels using Microsoft Office Picture Manager. WSxM software was used to estimate the root mean square and average of heights (Ra) expressed in μm and volumetric changes (μm ^3^ ), which can be considered precise indices of surface wear. 25

## Microhardness Measurement

Surface microhardness of the samples was calculated utilizing a Digital Display Vickers' Microhardness Tester (Model HVS-50, Laizhou Huayin Testing Instrument Co., Ltd., China) with a Vickers' diamond indenter and a 20X objective lens. The specimens' surfaces were loaded with 100-g for 15 seconds. Three indentations were created on the surface of each sample, uniformly distributed over a circle and at least 0.5 mm apart. The diagonal length of the indentations was calculated via an integrated calibrated microscope. Vickers' numbers were transformed into microhardness values, which were calculated utilizing the subsequent equation: HV = 1.854 P/d2, HV is Vickers' hardness in kgf/mm ^2^ , P is the load in kgf, and d is the length of the diagonals in mm. 26

## Statistical Analysis

Data processing and statistical analysis were performed utilizing the Statistical Package for Social Sciences (SPSS) version 20. Numerical data were described by using the mean, standard deviation, median, and range. Data normality was checked by inspecting the data distribution and performing Kolmogorov–Smirnov and Shapiro–Wilk tests. Kruskal–Wallis test was used to compare percent change between groups.

## Results

### Characterization of FG

**Fig. 1:**
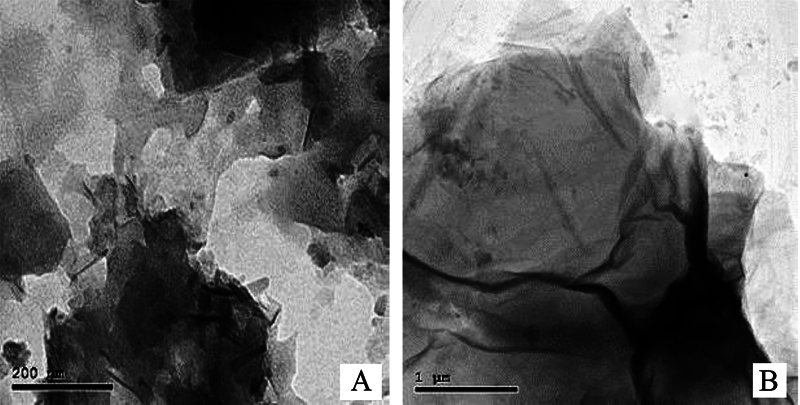
( **A** ) Transmission electron microscope (TEM) image of fluorinated graphene oxide nanosheets. ( **B** ) TEM image of fluorinated graphene oxide nanosheets at higher magnification.

The layered structural morphology of FG was studied using TEM, which demonstrated a thin transparent two-dimensional FG nanostructure with lateral dimensions between 200 nm and 2 μm; the surface appeared wrinkled with many ripples similar to graphene ( Fig. 1 ). According to the XRD pattern, FG disappeared on crystal face (002) while reinforced on crystal face (001), which revealed that FG was properly exfoliated and suggested the presence of a hexagonal crystal layer with an abundant fluorine concentration.

### Wear Resistance and Surface Roughness

**Fig. 2:**
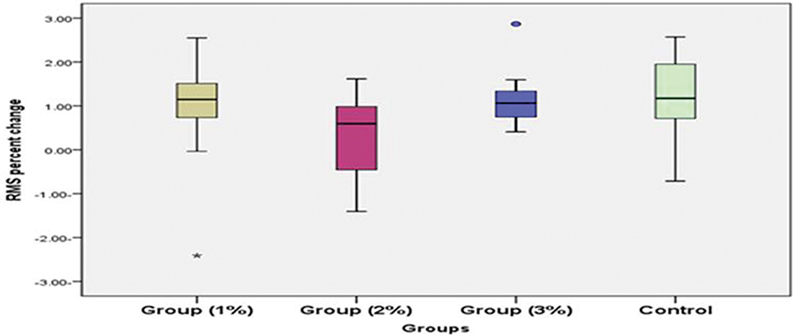
Boxplot illustrating median value of percent increase in root mean square (RMS) (%) in different groups.

**Fig. 3:**
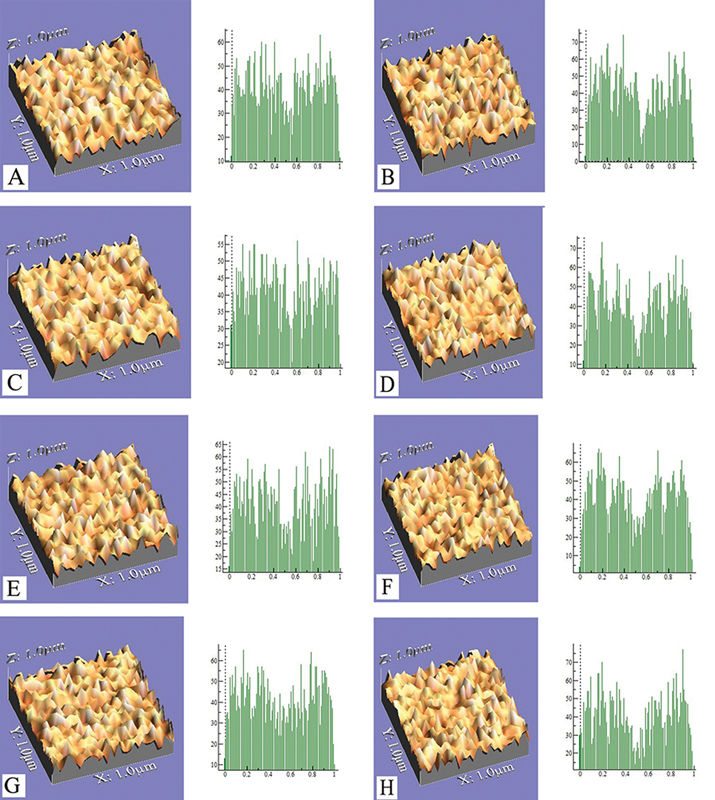
Three-dimensional images represent one sample from each group; ( **A, C, E** ) and ( **G** ) represent control and 1, 2,3 wt% fluorinated graphene/glass ionomer cements (FG/GIC) samples before brushing wear test, respectively. ( **B, D, F** ) and ( **H** ) represent control and 1,2,3 wt% FG/GIC samples after brushing wear test. Surface irregularities in ( **D, F** ) appeared with low heights and pits while ( **B, H** ) showed that the surface irregularities were larger and more pointed.

The results revealed that surface roughness of glass ionomer was increased after tooth brushing wear testing for experimental and control groups, with a statistically significant difference except for the 2wt% FG group, where the difference was not statistically significant ( Fig. 2 ). The 1 wt% FG group showed the lowest mean value of surface roughness after brushing wear test (0.2934 ± 0.003). While the 3wt% FG group showed the highest mean value (0.2969 ± 0.002), followed by the control group (0.2960 ± 0.002) and the 2 wt% FG group (0.2954 ± 0.002; Fig. 3 ), posthoc test revealed that the median value of percent increase in surface roughness in group 1 wt% FG was significantly lower than that in group 3wt% FG and the control group; additionally the highest median value of percent increase (median = 1.174, range [−0.71 to 2.57]) was recorded in control group, while the least percent increase value was recorded in group 2 wt% FG (median = 0.594, range [−1.141 to 1.62]). However, there was no statistically significant difference between the groups ( *p* = 0.189; Table 1 , Fig. 2 ).

**Table 1:** Descriptive statistics and comparison between groups regarding roughness (µm). ANOVA test and percent change in roughness (%) (Kruskal-Wallis test)

RMS		Mean	SD	95% Confidence interval for mean		Median	Min	Max	F	*p* -Value
				Lower bound	Upper bound					
Before	Group (1%)	0.2908 ^b^	0.003	0.289	0.293	0.2905	0.29	0.30	4.578	0.007*
	Group (2%)	0.2945 ^a^	0.002	0.293	0.296	0.2944	0.29	0.30		
	Group (3%)	0.2935 ^a^	0.002	0.292	0.295	0.2942	0.29	0.30		
	Control	0.2926 ^a,b^	0.003	0.291	0.295	0.2932	0.29	0.30		
After	Group (1%)	0.2934 ^y^	0.003	0.292	0.295	0.2937	0.29	0.30	5.971	0.002*
	Group (2%)	0.2954 ^x,y^	0.002	0.294	0.296	0.2956	0.29	0.30		
	Group (3%)	0.2969 ^x^	0.002	0.296	0.298	0.2965	0.29	0.30		
	Control	0.2960 ^x^	0.002	0.295	0.297	0.2962	0.29	0.30		
Percent change (%)	Group (1%)	0.9236	1.240	0.136	1.711	1.148	−2.41	2.55	—	0.189 nanoseconds
	Group (2%)	0.3200	0.992	-0.310	0.951	0.594	−1.41	1.62		
	Group (3%)	1.1521	0.634	0.749	1.555	1.064	0.41	2.87		
	Control	1.1622	1.053	0.493	1.831	1.174	−0.71	2.57		

Abbreviations: ANOVA, analysis of variance; SD, standard deviation.

Significance level *p* ≤0.05, * significant, ns = non-significant.

Posthoc test: Within the same comparison, means with different superscript letter are significantly different.

Regarding the results of volumetric changes (μm3) after tooth brushing test, the 3 wt% FG group showed the statistically significant highest values of volume loss (2.295 ± 1.243) ( *p* = 0.001) followed by the control (1.004 ± 1.609), group 2wt % (0.248 ± 1.428), and group 1wt% which recorded the least values (0.054 ± 1.178).

### Microhardness

The 1wt% FG group showed the highest mean value (49.23 ± 0.56), followed by group 2wt% (49.07 ± 0.67) and group 3wt% (47.75 ± 0.52). Further, the least value was reported in the control group (47.69 ± 0.74). Analysis of variance and posthoc tests revealed that groups 1wt% and 2wt% FG recorded statistically significant higher values than the 3wt% FG and control groups ( *p* = 0.000). While the difference between the 1wt% and 2wt% FG groups was not statistically significant, the difference between the 3 wt%FG and control groups was not statistically significant ( Table 2 ).

**Table 2:** Descriptive statistics and comparison between groups regarding microhardness (HV) (ANOVA test)

HV		Mean	SD	95% confidence interval for mean		Min	Max	F	*p* -Value
				Lower bound	Upper bound				
	Group (1%)	49.23 ^a^	0.56	48.87	49.58	48.40	49.85	20.68	0.000*
	Group (2%)	49.07 ^a^	0.67	48.64	49.49	47.90	49.90		
	Group (3%)	47.75 ^b^	0.52	47.42	48.09	47.07	48.60		
	Control	47.69 ^b^	0.74	47.22	48.16	46.20	48.60		

Abbreviations: ANOVA, analysis of variance; SD, standard deviation.

Significance level p≤0.05, * significant, ns = nonsignificant.

Post hoc test: means sharing the same superscript letter are not significantly different.

## Discussion

Many earlier efforts aimed to enhance the excessive brittleness, sensitivity to moisture, and reduced physical and mechanical characteristics of GICs by incorporating strengthening fillers into GIC matrix. 27 In the current research, we intended to enhance conventional GIC weaknesses by incorporating FG nanosheets.

GO was used to prepare FG by a hydrothermal process, 28 which provided safety and is regarded as a relatively simple process with minimal energy consumption. FG was produced as a white opaque powder that, after being incorporated into GIC, becomes yellowish, resulting in better esthetics. 29 30 TEM images of GO revealed that it is a nanosheet with a two-dimensional structure and an overall dimension of a few hundred nanometers. GO had an average thickness of nearly 0.702 nm. TEM images of FG showed slightly darker areas that could be related to FG nanosheets overlapping and stacking as shown in Fig. 1 . As compared with GO, FG had a higher value for the ratio of the intensity of the D- and G-Raman peaks (ID/IG) (1.05), while that of GO was (1.02) demonstrating that the atomic structure of FG is more disorganized. This is caused by the inclusion of many F atoms and the substitution of F atoms for oxygen atoms in the graphene network. 30

The results of TEM in an earlier study by Liu et al displayed that fluorine was properly distributed throughout the graphene network via hydrothermal process. By using TEM of the fracture surfaces, the distribution of FG sheets across GICs was assessed. The TEM image revealed that FG nanosheets were evenly distributed in the GIC matrix, proving that the FG has an excellent consistency with the GIC phase. ^30^ According to the findings of the wear resistance test in this study, adding FG to glass ionomer enhanced the wear resistance in all percentage; however, adding 1wt% FG to the GIC has the least surface roughness, volumetric changes, and the highest wear resistance. The improvement in mechanical properties could be related to many factors, including the high Young's modulus and inherent strength of FG and the consistent dispersion of the FG nanosheets through the GIC phase, which serves a significant role in GIC strengthening. 27 The relatively high particular surface area of FG and its two-dimensional structure facilitated the mechanical entanglement with the matrix, which resulted in load transmission to the FG nanosheet from the matrix when a crack developed due to a difference in the elastic modulus. 31

In addition, it seems that FG filled the gaps in the GIC matrix, the surface asperities were eliminated, and particles were smoothed out, which augmented the wear resistance of the FG/GIC combinations. Moreover, addition of FG to GIC turns GIC yellowish, thus improving the aesthetics. 27 30 31 32 33 The surface profile three-dimensional digital photographs of the samples in this study validated the wear resistance test findings as shown in Fig. 3 . The surface irregularities increased, became quite obvious and prominent after tooth brushing wear test for all groups ( Fig. 3B, D, F, H ). In the GIC 1wt% and 2wt% FG groups, the surface irregularities showed low height and pits with a crater-like appearance ( Fig. 3D, F ). While in the control and GIC 3wt% FG groups, the surface irregularities are larger, pointed, and seen with severe protrusions ( Fig. 3B, H ).

The outcomes in this study were in line with other studies. Sun et al in 2018 reported that the mechanical and physical characteristics of cementation materials could be improved by graphene and its derivatives. According to Kanwal et al, the fracture resistance and strength of 0.5 wt% graphene/bioactive glass compounds raised significantly by 38%. Additionally, Dubey et al deduced that adding GO to Mineral Trioxide Aggregate (MTA) paste could improve both compressive and tensile strength. Moreover, Zhou et al investigated the tribological behavior of the poly-imide/FG combinations and proved that the addition of FG greatly enhanced the tribological behavioral quality of poly-imide. 12 21 22 24

The microhardness test revealed that GIC (1wt% and 2wt %) FG groups recorded significantly higher values than 3wt% FG and control groups. This could be due to the impact of both graphene and its derivatives on the improvement of mechanical strength and hardness, which may be attributed to the crack propagation process, that was explored in earlier studies. 18 24 34

In this study, the GIC 3wt% FG group showed lower Vickers Hardness number (VHN) and wear resistance values compared with the other tested groups. This could be related to the increased concentration of FG as explained in a previous study which showed that at higher concentrations of FG, carbon-based compounds can aggregate within the cement matrix causing incomplete setting. 24 Similarly, excessive FG sheets incorporation into the matrix represented an obstacle for their homogenous dispersion causing aggregation, which prevented the cement matrix hydration, causing clustering of the cement. Moreover, voids produced by these agglomerates turned into weak spots within the cement matrix. 35 36 Consequently, these spots resulted in decreased hardness values. So, incorporation of FG should only be in a restricted percentage. However, adding FG could generally increase the strength of GICs. 37 38

Based on this study observations, GIC (3wt %) FG samples showed delayed setting reaction compared with the conventional GIC and GIC (1wt%, 2 wt %) FG. The high concentration of FG was found to hinder the setting process of GIC. Nineteen prior studies had demonstrated that compounds based on carbon can aggregate within the cement matrix and alter AL/Si ratio, leading to a delayed setting at higher concentrations. 39

In conclusion, the FG with a white color was uniformly distributed in the GIC matrix. It has the potential to enhance the mechanical properties and wear resistance of glass ionomer. The GIC (1wt %) FG showed superior hardness and better wear resistance with maintenance of good handling properties and setting procedure. The null hypothesis could be accepted, so modified GIC by FG nanosheets appeared to be a promising dental restoration that may exhibit improved clinical performance.
